# Chronic Administrations of Guanfacine on Mesocortical Catecholaminergic and Thalamocortical Glutamatergic Transmissions

**DOI:** 10.3390/ijms22084122

**Published:** 2021-04-16

**Authors:** Kouji Fukuyama, Tomosuke Nakano, Takashi Shiroyama, Motohiro Okada

**Affiliations:** Department of Neuropsychiatry, Division of Neuroscience, Graduate School of Medicine, Mie University, Tsu 514-8507, Japan; k-fukuyama@clin.medic.mie-u.ac.jp (K.F.); t-nakano@clin.medic.mie-u.ac.jp (T.N.); takashi@clin.medic.mie-u.ac.jp (T.S.)

**Keywords:** attention-deficit/hyperactivity disorder, guanfacine, dopamine, norepinephrine, L-glutamate, GABA, α2A adrenoceptor

## Abstract

It has been established that the selective α2A adrenoceptor agonist guanfacine reduces hyperactivity and improves cognitive impairment in patients with attention-deficit/hyperactivity disorder (ADHD). The major mechanisms of guanfacine are considered to involve the activation of the postsynaptic α2A adrenoceptor of glutamatergic pyramidal neurons in the frontal cortex, but the effects of chronic guanfacine administration on catecholaminergic and glutamatergic transmissions associated with the orbitofrontal cortex (OFC) are yet to be clarified. The actions of guanfacine on catecholaminergic transmission, the effects of acutely local and systemically chronic (for 7 days) administrations of guanfacine on catecholamine release in pathways from the locus coeruleus (LC) to OFC, the ventral tegmental area (VTA) and reticular thalamic-nucleus (RTN), from VTA to OFC, from RTN to the mediodorsal thalamic-nucleus (MDTN), and from MDTN to OFC were determined using multi-probe microdialysis with ultra-high performance liquid chromatography. Additionally, the effects of chronic guanfacine administration on the expression of the α2A adrenoceptor in the plasma membrane fraction of OFC, VTA and LC were examined using a capillary immunoblotting system. The acute local administration of therapeutically relevant concentrations of guanfacine into the LC decreased norepinephrine release in the OFC, VTA and RTN without affecting dopamine release in the OFC. Systemically, chronic administration of therapeutically relevant doses of guanfacine for 14 days increased the basal release of norepinephrine in the OFC, VTA, RTN, and dopamine release in the OFC via the downregulation of the α2A adrenoceptor in the LC, OFC and VTA. Furthermore, systemically, chronic guanfacine administration did not affect intrathalamic GABAergic transmission, but it phasically enhanced thalamocortical glutamatergic transmission. The present study demonstrated the dual actions of guanfacine on catecholaminergic transmission—acute attenuation of noradrenergic transmission and chronic enhancement of noradrenergic transmission and thalamocortical glutamatergic transmission. These dual actions of guanfacine probably contribute to the clinical effects of guanfacine against ADHD.

## 1. Introduction

Attention deficit hyperactivity disorder (ADHD) is a common neurodevelopmental and neuropsychiatric disorder in children and adolescents (estimated prevalence of ADHD in adults is 5%) [1,2]. Usually, ADHD onsets in childhood and persists to adulthood. Sometimes onset occurs during adolescence [2]. Adult-onset ADHD is characterized by dysregulations of emotion, attention, executive function, impulsivity, and restlessness, which result in several socio-psychological disturbances [2].

The treatment of ADHD usually combines pharmacotherapy and behavioural therapy [2]. In pharmacotherapy, it has been established that stimulant class agents (methylphenidate and amphetamines) are the first-line agents, since these show the most effectiveness [3,4]. Unfortunately, stimulant class agents are probably not effective in up to 30% of patients with ADHD [5], and they are possibly associated with several adverse responses, including dependency or diversion [6]. Therefore, non-stimulant class medications need to be used as an adjunctive therapy or as a monotherapy alternative in patients with suboptimal responses to or intolerable side-effects from stimulants [6,7]. The availability of different mechanisms of medications provides opportunities for additional/augmentation medication strategies when ADHD symptoms cannot be adequately improved by prior therapies, such as stimulant class agents [7].

Guanfacine is an approved agent for the treatment of ADHD as a non-stimulant class agent in Japan, the USA and Europe [1,2,3,4]. Clinical trials indicate that guanfacine is effective for the treatment of ADHD in children and adolescents [7]. Notably, guanfacine improves several symptoms of ADHD, including oppositional symptoms [8]. Compared to atomoxetine, guanfacine may contribute to greater control of ADHD and rapid onset of efficacy [7]. Furthermore, a meta-analysis study reported a higher incidence of events of suicidal ideation induced by atomoxetine within one month of starting atomoxetine [9]; however, there is no reported risk of suicidal ideation with guanfacine [7,10].

Guanfacine is a selective agonist of the α2A adrenoceptor [11,12,13,14]. The α2A adrenoceptor is mainly expressed in the dendritic spines of frontal glutamatergic pyramidal neurones [13]. Based on the findings, the major mechanisms of action of guanfacine are proposed as two hypotheses [7,14,15]. The first hypothesis is that guanfacine activates frontal pyramidal neurones associated with working memory due to blockades of the hyperpolarisation-activated cyclic nucleotide-gated channel (HCN) [16], induced by the activation of the postsynaptic α2A adrenoceptor in superficial layers (HCN hypothesis) [14]. The second hypothesis is that guanfacine suppresses the hyper-function of pyramidal neurones of ADHD due to an enhanced inhibitory postsynaptic α2A adrenoceptor (excitatory postsynaptic current hypothesis) [7,15]. These two hypotheses emphasise the importance of the intra-frontal glutamatergic system. Both hypotheses were supported by a number of experiments. In particular, both guanfacine and clonidine improve attention/cognition performance and the regulation of impulsivity in rat ADHD models [17], but do not improve behaviour in the α2A adrenoceptor knockout model [18]. The behavioural effects of both guanfacine and clonidine were attenuated by α2A adrenoceptor antagonists but were unaffected by antagonists of α2B or α2C adrenoceptors [17]. These preclinical findings suggest that the modulation of noradrenergic transmission via the activation of the α2A adrenoceptor probably plays fundamental roles in the pathophysiology of ADHD.

Clinical imaging studies revealed that the functional disturbance associated with the orbitofrontal cortex (OFC) [19], ventral tegmental area (VTA) [20] and mediodorsal thalamic nucleus (MDTN) [21] contributed to cognitive dysfunction in patients with ADHD. The locus coeruleus (LC), the most dominant noradrenergic nucleus, regulates cognition/perception via the projection of noradrenergic terminals to various brain regions, including the OFC and reticular thalamic nucleus (RTN) [22,23]. Therefore, to further understand the pathophysiology of ADHD, the effects of acute and chronic administrations of guanfacine on noradrenergic transmission in the frontal cortex and outside of the cortex should be clarified. Acute guanfacine administration in particular decreased releases of norepinephrine in the LC and OFC, and dopamine in the OFC, whereas the subchronic administration of a therapeutically relevant dose of guanfacine (for 7 days) suppressed the inhibitory effects of the α2A adrenoceptor but decreased basal releases of norepinephrine and dopamine in the OFC [24]. The mechanisms of suppression of the inhibitory effects of the α2A adrenoceptor induced by subchronic administration of guanfacine are speculated to cause the desensitisation or downregulation of the α2A adrenoceptor. However, neither the kinetics of desensitisation nor the downregulation of the α2A adrenoceptor induced by guanfacine has been clarified [24]. 

The OFC receives catecholaminergic projections, including the selective dopaminergic pathway from the VTA and selective noradrenergic and dopamine/norepinephrine co-releasing pathways from the LC [25,26,27,28]. The selective dopaminergic and noradrenergic terminals receive GABAergic inhibitory inputs in the deeper layers of the OFC, whereas dopamine/norepinephrine co-releasing terminals from the LC to the superficial layers of the OFC receive stimulatory α-amino-3-hydroxy-5-methyl-4-isoxazolepropionic acid (AMPA)/glutamatergic regulation from the MDTN [27,28]. Glutamatergic neurones in the MDTN are also regulated by GABAergic inhibition from the RTN, which receives excitatory mesothalamic noradrenergic inputs via the excitatory α1 adrenoceptor [29]. Based on these anatomical findings regarding catecholaminergic pathways, to explore the mechanisms of chronic administration of guanfacine, the present study determined the effects of acute and chronic (for 14 days) administration of therapeutically relevant doses of guanfacine on the catecholaminergic transmission in LC-OFC, LC-VTA-OFC, and glutamatergic transmission in LC-RTN-MDTN-OFC using multiprobe microdialysis, as well as α2A adrenoceptor expression in the plasma membranes of LC, VTA and OFC using capillary immunoblotting systems.

## 2. Results

### 2.1. Effects of Acute and Chronic Administration of Therapeutic-Relevant Dose of Guanfacine on Extracellular Levels of Norepinephrine and Dopamine in the OFC and VTA (Study 1)

To determine the effects of local administrations of guanfacine (200 nM) into the LC [24] on extracellular levels of norepinephrine and dopamine in the OFC, after the systemically chronic administration of therapeutic-relevant dose of guanfacine (0 or 0.12 mg/kg/day) [17,24,30] for 14 days using osmotic pump (2002, Alzet, Cupertino, CA, USA) (N = 48), microdialysis probes were inserted into the LC, VTA, RTN, MDTN and OFC [27,28,31,32,33]. After the chronic guanfacine-free administration for 14 days, the perfusate in the LC was switched from MRS to MRS containing guanfacine (200 nM) (acute effects). After the systemically chronic administration of guanfacine (0.12 mg/kg/day) for 14 days, the perfusate in LC was switched from MRS to MRS containing guanfacine (200 nM) (chronic effects).

#### 2.1.1. Effects of Acute and Chronic Administration of Therapeutic-Relevant Dose of Guanfacine on Extracellular Norepinephrine Level in the OFC

**Figure 1:**
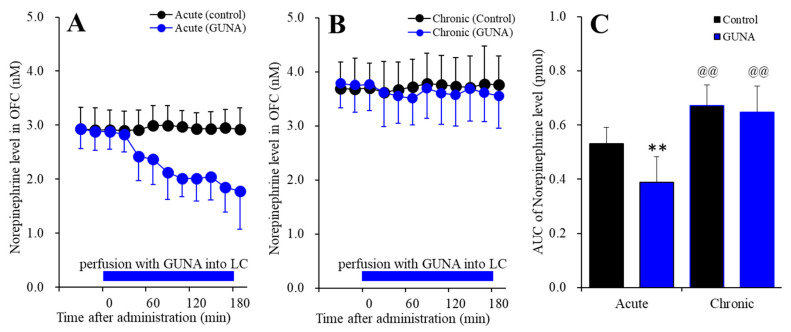
Effects of acutely local administration (perfusion with) 200 nM guanfacine into the locus coeruleus (LC) on extracellular norepinephrine level in the orbitofrontal cortex (OFC), after the systemically chronic administration of guanfacine (0 or 0.12 mg/kg/day for 14 days). Panel (**A**) indicates the effects of perfusion with 200 nM guanfacine into the LC after chronic administration of guanfacine (0 mg/kg/day) for 14 days (acute effects). Panel (**B)** indicates effects of 200 nM guanfacine after systemically chronic administration of guanfacine (0.12 mg/kg/day) for 14 days (chronic effects). Ordinates: mean ± SD (n = 6) of extracellular norepinephrine level (nM), abscissa: time after administration of perfusion with guanfacine into the LC (min). Blue bars: perfusion of 200 nM guanfacine into LC. Panel (**C**) indicates the AUC_20–180 min_ values of extracellular norepinephrine level during perfusion with guanfacine (from 20 to 180 min). Ordinates: mean ± SD (n = 6) of AUC_20–180 min_ values of extracellular norepinephrine level (pmol). Black and blue columns indicate respective control and perfusion of guanfacine of AUC values of norepinephrine levels. When the effects of guanfacine were statistically significant compared using multivariate analysis of variance (MANOVA), the data were analysed using Tukey’s post hoc test. ** *p* < 0.01, compared with control. @@ *p* < 0.01: compared with acute effects of guanfacine.

Perfusion with guanfacine into the LC, after systemically chronic administration (0 or 0.12 mg/kg/day guanfacine for 14 days), affected extracellular norepinephrine level in the OFC [F_time_(5.6,113.0) = 7.0 (*p* < 0.01), F_administration_(1,20) = 33.4 (*p* < 0.01), F_guanfacine_(1,20) = 4.8 (*p* < 0.05), F_guanfacine*time_(5.6,113.0) = 11.1 (*p* < 0.01), F_guanfacine*adminisdtration_(1,20) = 2.6 (*p* > 0.1), F_adminisdtration*time_(5.6,113.0) = 7.9 (*p* < 0.01), F_guanfacine*administration*time_(5.6,113.0) = 5.7 (*p* < 0.01)] (Figure 1). Acute administration of guanfacine (perfusion with 200 nM guanfacine into the LC) decreased extracellular norepinephrine in the OFC (Figure 1A), whereas after chronic guanfacine administration, perfusion with 200 nM guanfacine into the LC did not have an effect (Figure 1B). Furthermore, chronic administration of guanfacine increased basal extracellular norepinephrine level in the OFC (Figure 1C). Therefore, chronic administration of therapeutic-relevant does of guanfacine (0.12 mg/kg/day for 14 days), prevented the acute inhibitory effects of guanfacine on the mesocortical (LC-OFC) noradrenergic transmission and enhances the mesocortical noradrenergic transmission.

#### 2.1.2. Effects of Acute and Chronic Administration of Therapeutic-Relevant Dose of Guanfacine on Extracellular Dopamine Level in the OFC

**Figure 2:**
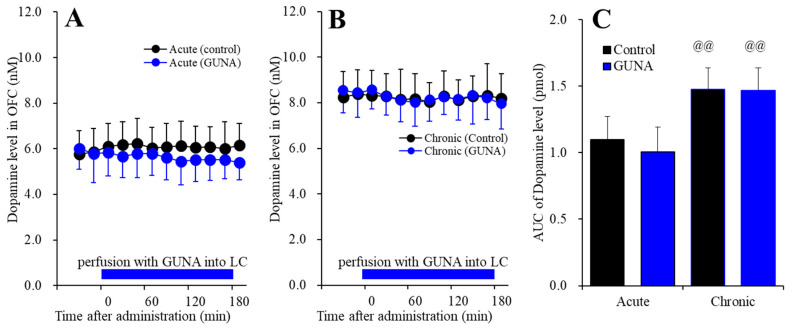
Effects of acute local administration (perfusion with) 200 nM guanfacine into the LC on extracellular dopamine level in the OFC, after the systemically chronic administration of guanfacine (0 or 0.12 mg/kg/day for 14 days). Panel (**A**) indicates the effects of perfusion with 200 nM guanfacine into the LC after chronic administration of guanfacine (0 mg/kg/day) for 14 days (acute effects). Panel (**B**) indicates effects of 200 nM guanfacine after systemically chronic administration of guanfacine (0.12 mg/kg/day) for 14 days (chronic effects). Ordinates: mean±SD (n = 6) of extracellular dopamine level (nM), abscissa: time after administration of perfusion with guanfacine into the LC (min). Blue bars: perfusion of 200 nM guanfacine into LC. Panel (**C**) indicates the AUC_20–180 min_ values of extracellular dopamine level during perfusion with guanfacine (from 20 to 180 min). Ordinates: mean ± SD (n = 6) of AUC_20–180 min_ values of extracellular dopamine level (pmol). Black and blue columns indicate respective control and perfusion of guanfacine of AUC values of dopamine levels. When the effects of guanfacine were statistically significant compared using MANOVA, the data were analysed using Tukey’s post hoc test. @@ *p* < 0.01: compared with acute effects of guanfacine.

Perfusion with guanfacine into the LC, after systemically chronic administration (0 or 0.12 mg/kg/day guanfacine for 14 days), affected extracellular dopamine level in the OFC [F_time_(9,180) = 1.3 (*p* > 0.1), F_administration_(1,20) = 36.3 (*p* < 0.01), F_guanfacine_(1,20) = 0.4 (*p* > 0.1), F_guanfacine*time_(9,180) = 1.4 (*p* > 0.1), F_guanfacine*adminisdtration_(1,20) = 0.3 (*p* > 0.1), F_adminisdtration*time_(9,180) = 1.3 (*p* > 0.1), F_guanfacine*administration*time_(9,180) = 0.5 (*p* > 0.1)] (Figure 2). Acute administration of guanfacine (perfusion with 200 nM guanfacine into the LC) did not affect extracellular dopamine in the OFC (Figure 2A), and after chronic guanfacine administration, perfusion with 200 nM guanfacine into the LC also had no effect (Figure 2B). However, the chronic administration of guanfacine increased the basal extracellular dopamine level in the OFC (Figure 2C). These results indicate the difference mechanisms of the effects of chronic administration of guanfacine on mesocortical noradrenergic and dopaminergic transmissions, since, similar to norepinephrine, chronic administration of therapeutically relevant does of guanfacine (0.12 mg/kg/day for 14 days) enhances the mesocortical dopaminergic transmission without acutely affecting extracellular dopamine level in the OFC. 

#### 2.1.3. Effects of Acute and Chronic Administration of Therapeutic-Relevant Dose of Guanfacine on Extracellular Norepinephrine Level in the VTA

**Figure 3:**
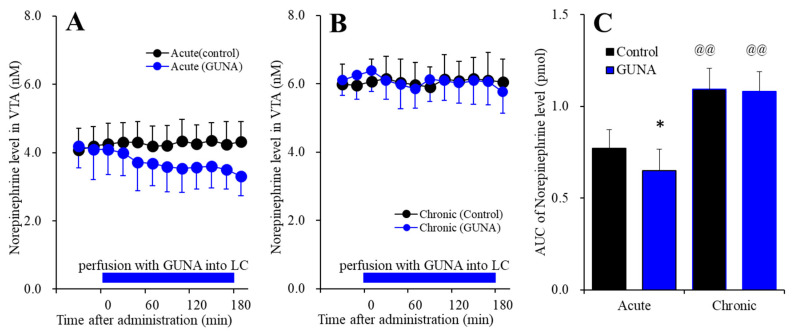
Effects of acutely local administration (perfusion with) 200 nM guanfacine into the LC on extracellular norepinephrine level in the VTA, after the systemically chronic administration of guanfacine (0 or 0.12 mg/kg/day for 14 days). Panel (**A**) indicates the effects of perfusion with 200 nM guanfacine into the LC after chronic administration of guanfacine (0 mg/kg/day) for 14 days (acute effects). Panel (**B**) indicates effects of 200 nM guanfacine after systemically chronic administration of guanfacine (0.12 mg/kg/day) for 14 days (chronic effects). Ordinates: mean±SD (n = 6) of extracellular norepinephrine level (nM), abscissa: time after administration of perfusion with guanfacine into the LC (min). Blue bars: perfusion of 200 nM guanfacine into LC. Panel (**C**) indicates the AUC20–180 min values of extracellular norepinephrine level during perfusion with guanfacine (from 20 to 180 min). Ordinates: mean±SD (n = 6) of AUC20–180 min values of extracellular norepinephrine level (pmol). Black and blue columns indicate respective control and perfusion of guanfacine of AUC values of norepinephrine levels. When the effects of guanfacine were statistically significant compared using MANOVA, the data were analysed using Tukey’s post hoc test. * *p* < 0.01, compared with control. @@ *p* < 0.01: compared with acute effects of guanfacine.

Perfusion with guanfacine into the LC, after systemically chronic administration (0 or 0.12 mg/kg/day guanfacine for 14 days), affected extracellular norepinephrine level in the VTA [F_time_(9,180) = 5.2 (*p* < 0.01), F_administration_(1,20) = 69.4 (*p* < 0.01), F_guanfacine_(1,20) = 1.7 (*p* > 0.1), F_guanfacine*time_(9,180) = 6.8 (*p* < 0.01), F_guanfacine*adminisdtration_(1,20) = 1.3 (*p* > 0.1), F_adminisdtration*time_(9,180) = 1.5 (*p* > 0.05), F_guanfacine*administration*time_(9,180) = 1.8 (*p* > 0.05)] (Figure 3). Acute administration of guanfacine (perfusion with 200 nM guanfacine into the LC) decreased extracellular norepinephrine in the VTA (Figure 3A), whereas after chronic guanfacine administration, perfusion with 200 nM guanfacine into the LC had no effect (Figure 3B). Furthermore, the chronic administration of guanfacine increased basal extracellular norepinephrine level in the VTA (Figure 3C). Therefore, similar to the LC-OFC noradrenergic pathway, the chronic administration of therapeutically relevant does of guanfacine (0.12 mg/kg/day for 14 days) prevented the acute inhibitory effects of guanfacine on the LC-VTA noradrenergic transmission, but enhanced the LC-VTA noradrenergic transmission.

#### 2.1.4. Effects of Acute and Chronic Administration of Therapeutic-Relevant Dose of Guanfacine on Extracellular Norepinephrine Level in the RTN

**Figure 4:**
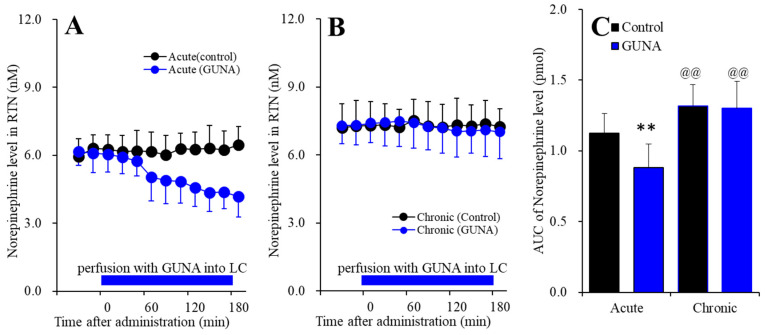
Effects of acutely local administration (perfusion with) 200 nM guanfacine into the LC on extracellular norepinephrine level in the RTN, after the systemically chronic administration of guanfacine (0 or 0.12 mg/kg/day for 14 days). Panel (**A**) indicates the effects of perfusion with 200 nM guanfacine into the LC after chronic administration of guanfacine (0 mg/kg/day) for 14 days (acute effects). Panel (**B**) indicates effects of 200 nM guanfacine after systemically chronic administration of guanfacine (0.12 mg/kg/day) for 14 days (chronic effects). Ordinates: mean ± SD (n = 6) of extracellular norepinephrine level (nM), abscissa: time after administration of perfusion with guanfacine into the LC (min). Blue bars: perfusion of 200 nM guanfacine into LC. Panel (**C**) indicates the AUC_20–180 min_ values of extracellular norepinephrine level during perfusion with guanfacine (from 20 to 180 min). Ordinates: mean±SD (n = 6) of AUC_20–180 min_ values of extracellular norepinephrine level (pmol). Black and blue columns indicate respective control and perfusion of guanfacine of AUC values of norepinephrine levels. When the effects of guanfacine were statistically significant compared using MANOVA, the data were analysed using Tukey’s post hoc test. ** *p* < 0.01, compared with control. @@ *p* < 0.01: compared with acute effects of guanfacine.

Perfusion with guanfacine into the LC, after systemically chronic administration (0 or 0.12 mg/kg/day guanfacine for 14 days), affected extracellular norepinephrine level in the RTN [F_time_(9,180) = 11.8 (*p* < 0.01), F_administration_(1,20) = 20.5 (*p* < 0.01), F_guanfacine_(1,20) = 3.2 (*p* > 0.05), F_guanfacine*time_(9,180) = 16.3 (*p* < 0.01), F_guanfacine*adminisdtration_(1,20) = 2.5 (*p* > 0.1), F_adminisdtration*time_(9,180) = 5.3 (*p* < 0.01), F_guanfacine*administration*time_(9,180) = 6.8 (*p* < 0.01)] (Figure 4). Acute administration of guanfacine (perfusion with 200 nM guanfacine into the LC) decreased extracellular norepinephrine in the RTN (Figure 4A), whereas after chronic guanfacine administration, perfusion with 200 nM guanfacine into the LC did not affect (Figure 4B). Furthermore, chronic administration of guanfacine increased basal extracellular norepinephrine level in the RTN (Figure 4C). Therefore, similar to LC-OFC and LC-VTA noradrenergic pathways, chronic administration of therapeutic-relevant does of guanfacine (0.12 mg/kg/day for 14 days), prevented the acute inhibitory effects of guanfacine on the LC-RTN noradrenergic transmission, but enhances the LC-RTN noradrenergic transmission.

#### 2.1.5. Effects of Acute and Chronic Administration of Therapeutically Relevant Dose of Guanfacine on Extracellular GABA Level in the MDTN

**Figure 5:**
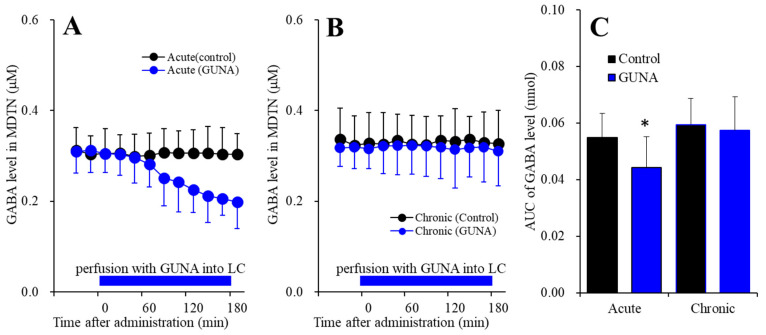
Effects of acutely local administration (perfusion with) 200 nM guanfacine into the LC on extracellular GABA level in the MDTN, after the systemically chronic administration of guanfacine (0 or 0.12 mg/kg/day for 14 days). Panel (**A**) indicates the effects of perfusion with 200 nM guanfacine into the LC after chronic administration of guanfacine (0 mg/kg/day) for 14 days (acute effects). Panel (**B**) indicates effects of 200 nM guanfacine after systemically chronic administration of guanfacine (0.12 mg/kg/day) for 14 days (chronic effects). Ordinates: mean±SD (n = 6) of extracellular GABA level (μM), abscissa: time after administration of perfusion with guanfacine into the LC (min). Blue bars: perfusion of 200 nM guanfacine into LC. Panel (**C**) indicates the AUC20–180 min values of extracellular GABA level during perfusion with guanfacine (from 20 to 180 min). Ordinates: mean ± SD (n = 6) of AUC20–180 min values of extracellular GABA level (pmol). Black and blue columns indicate respective control and perfusion of guanfacine of AUC values of GABA levels. When the effects of guanfacine were statistically significant compared using MANOVA, the data were analysed using Tukey’s post hoc test. * *p* < 0.05, compared with control.

Perfusion with guanfacine into the LC, after systemically chronic administration (0 or 0.12 mg/kg/day guanfacine) for 14 days, affected extracellular GABA level in the MDTN [F_time_(7.3,144.5) = 6.6 (*p* < 0.01), F_administration_(1,20) = 4.0 (*p* > 0.05), F_guanfacine_(1,20) = 1.9 (*p* > 0.1), F_guanfacine*time_(7.3,144.5) = 7.7 (*p* < 0.01), F_guanfacine*adminisdtration_(1,20) = 0.8 (*p* > 0.1), F_adminisdtration*time_(7.3,144.5) = 5.8 (*p* < 0.01), F_guanfacine*administration*time_(7.3,144.5) = 5.5 (*p* < 0.01)] (Figure 5). Acute administration of guanfacine (perfusion with 200 nM guanfacine into the LC) decreased extracellular GABA in the MDTN (Figure 5A), whereas after chronic guanfacine administration, perfusion with 200 nM guanfacine into the LC had no effect (Figure 5B). Contrary to catecholamine, the chronic administration of guanfacine did not affect basal extracellular GABA level in the MDTN (Figure 5C). The GABAergic neurons receive excitatory noradrenergic inputs via postsynaptic α1 adrenoceptor [24,34,35]. The reduction in GABA level in the MDTN by local administration of guanfacine into the LC is generated by reduced norepinephrine release in the RTN. Contrary to acute response, the increased extracellular norepinephrine level cannot produce the enhancement of GABAergic inputs to the MDTN, which suggests that excitatory noradrenergic transmission to the RTN probably affects phasic activation [24,35]. 

### 2.2. Effects of Local Administration of Prazosine into the VTA on Extracellular Levels of Dopamine in the OFC of Chronic Guanfacine Administration (Study 2)

To determine the effects of local administrations of α1 adrenoceptor antagonist, 100 μM prazosin (PRZ) [27,28,36,37] into the VTA on extracellular dopamine level in the OFC, during the systemically chronic administration of guanfacine (0 or 0.12 mg/kg/day for 14 days) using osmotic pump (N = 24), microdialysis probes were inserted into the VTA and OFC. During the chronic guanfacine-free administration for 14 days, the perfusate in the VTA was switched from MRS to MRS containing PRZ (100 μM) (acute effects). During the systemically chronic administration of guanfacine (0.12 mg/kg/day) for 14 days, the perfusate in VTA was switched from MRS to MRS containing PRZ (100 μM) (chronic effects).

**Figure 6:**
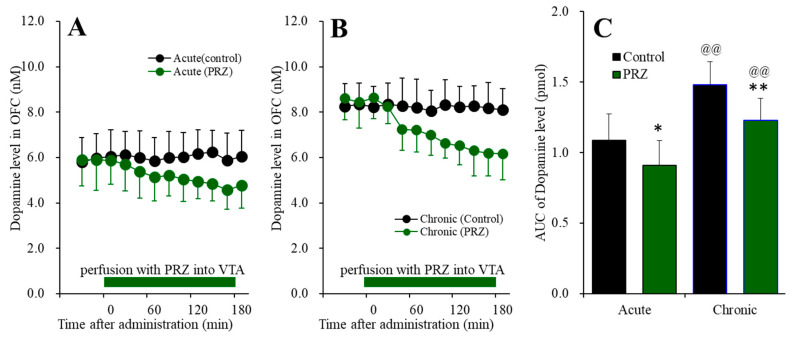
Effects of acutely local administration (perfusion with) 100 μM prazosin (PRZ) into the VTA on extracellular dopamine level in the OFC, after the systemically chronic administration of guanfacine (0 or 0.12 mg/kg/day for 14 days). Panel (**A**) indicates the effects of perfusion with 100 μM PRZ into the VTA after chronic administration of guanfacine (0 mg/kg/day) for 14 days (acute effects). Panel (**B**) indicates effects of 100 μM PRZ after systemically chronic administration of guanfacine (0.12 mg/kg/day) for 14 days (chronic effects). Ordinates: mean ± SD (n = 6) of extracellular dopamine level (nM), abscissa: time after administration of perfusion with PRZ into the VTA (min). Green bars: perfusion of 100 μM PRZ into VTA. Panel (**C**) indicates the AUC_20–180 min_ values of extracellular dopamine level during perfusion with PRZ (from 20 to 180 min). Ordinates: mean±SD (n = 6) of AUC_20–180 min_ values of extracellular dopamine level (pmol). Black and green columns indicate respective control and perfusion of PRZ of AUC values of dopamine levels. When the effects of PRZ were statistically significant compared using MANOVA, the data were analysed using Tukey’s post hoc test. * *p* < 0.05, ** *p* < 0.01, compared with control. @@ *p* < 0.01: compared with acute effects of PRZ.

Perfusion with 100 μM PRZ into the VTA, during systemically chronic administration (0 or 0.12 mg/kg/day guanfacine) for 14 days, affected extracellular dopamine level in the OFC [F_time_(6.0,119.2) = 20.2 (*p* < 0.01), F_administration_(1,20) = 26.9 (*p* < 0.01), F_guanfacine_(1,20) = 7.3 (*p* < 0.05), F_guanfacine*time_(6.0,119.2) = 19.2 (*p* < 0.01), F_guanfacine*adminisdtration_(1,20) = 0.2 (*p* > 0.1), F_adminisdtration*time_(6.0,119.2) = 3.5 (*p* < 0.01), F_guanfacine*administration*time_(6.0,119.2) = 1.9 (*p* > 0.05)] (Figure 6). Perfusion with 100 μM PRZ into the VTA decreased extracellular dopamine in the OFC, and after chronic guanfacine administration, perfusion with 100 μM PRZ into the VTA also decreased (Figure 6A,B). Furthermore, the chronic administration of guanfacine increased basal extracellular dopamine level in the OFC (Figure 6C). These results suggest that the responses of dopaminergic neurons in the VTA to the excitatory noradrenergic inputs from the LC are not affected by chronic guanfacine administration, whereas the elevation of basal extracellular dopamine level in the OFC was induced by chronic guanfacine administration.

### 2.3. Effects of Local Administration of AMPA into the MDTN on Extracellular Levels of Norepinephrine, Dopamine and L-glutamate in the OFC of Chronic Guanfacine Administration (Study 3)

To determine the effects of local administrations of AMPA/glutamate receptor agonist, 100 μM AMPA into the MDTN on extracellular levels of norepinephrine, dopamine and L-glutamate in the OFC, after the systemically chronic administration of guanfacine (0 or 0.12 mg/kg/day) for 14 days using osmotic pump (N = 12), microdialysis probes were inserted into the MDTN and OFC. During the chronic guanfacine-free administration for 14 days, the perfusate in the MDTN was switched from MRS to MRS containing 100 μM AMPA (acute effects). During the systemically chronic administration of guanfacine (0.12 mg/kg/day) for 14 days, the perfusate in MDTN was switched from MRS to MRS containing AMPA (100 μM) (chronic effects).

**Figure 7:**
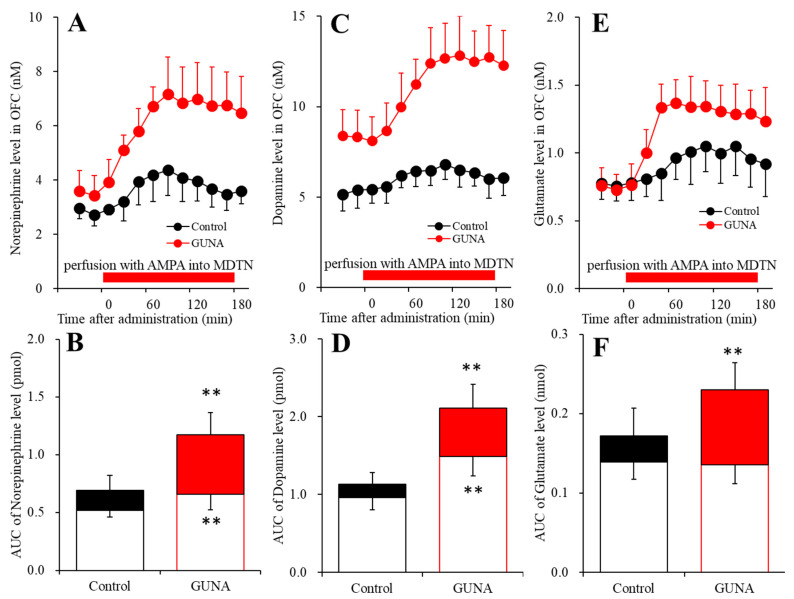
Effects of acutely local administration (perfusion with) 100 μM α-amino-3-hydroxy-5-methyl-4-isoxazolepropionic acid (AMPA) into the MDTN on extracellular levels of norepinephrine, dopamine and L-glutamate in the OFC, after the systemically chronic administration of guanfacine (0 or 0.12 mg/kg/day for 14 days). Panels (**A**,**C**,**E**) indicate the effects of perfusion with 100 μM AMPA into the MDTN after chronic administration of guanfacine (0 or 0.12 mg/kg/day) for 14 days on extracellular levels of norepinephrine, dopamine and L-glutamate in the OFC, respectively. Ordinates: mean±SD (n = 6) of extracellular levels of norepinephrine (nM), dopamine (nM) or L-glutamate (μM), abscissa: time after administration of perfusion with AMPA into the MDTN (min). Red bars: perfusion of 100 μM AMPA into the MDTN. Panels (**B**,**D**,**F**) indicate the AUC20–180 min values of extracellular levels of norepinephrine, dopamine and L-glutamate during perfusion with AMPA (from 20 to 180 min). Ordinates: mean ± SD (n = 6) of AUC20–180 min values of extracellular levels of norepinephrine (pmol), dopamine (pmol) and L-glutamate (nmol), respectively. Opened, black and red columns indicate AUC vales during pre-AMPA and AMPA-evoked releases control and chronic guanfacine administrations, respectively. When the effects of AMPA were statistically significant compared using MANOVA, the data were analysed using Tukey’s post hoc test. ** *p* < 0.01, compared with control.

Perfusion with 100 μM AMPA into the MDTN, during systemically chronic administration (0 or 0.12 mg/kg/day guanfacine) for 14 days, affected extracellular norepinephrine level in the OFC [F_time_(7.9,79.4) = 44.8 (*p* < 0.01), F_administration_(1,10) = 23.8 (*p* < 0.01), F_adminisdtration*time_(7.9,79.4) = 11.7 (*p* < 0.01)] (Figure 7A,B). Perfusion with 100 μM AMPA into the MDTN increased extracellular norepinephrine in the OFC (Figure 7A,B). Chronic guanfacine administration enhanced 100 μM AMPA-evoked norepinephrine release in the OFC (Figure 7A,C). Furthermore, chronic administration of guanfacine increased basal extracellular norepinephrine level in the OFC (Figure 7A,B). 

Perfusion with 100 μM AMPA into the MDTN, during systemically chronic administration (0 or 0.12 mg/kg/day guanfacine) for 14 days, affected extracellular dopamine level in the OFC [F_time_(9,90) = 57.1 (*p* < 0.01), F_administration_(1,10) = 45.5 (*p* < 0.01), F_adminisdtration*time_(9,90) = 25.6 (*p*<0.01)] (Figure 7C,D). Perfusion with 100 μM AMPA into the MDTN increased extracellular dopamine in the OFC (Figure 7C,D). Chronic guanfacine administration enhanced 100 μM AMPA-evoked dopamine release in the OFC (Figure 7C,D). Furthermore, the chronic administration of guanfacine increased basal extracellular dopamine level in the OFC (Figure 7C,D).

Perfusion with 100 μM AMPA into the MDTN, during systemically chronic administration (0 or 0.12 mg/kg/day guanfacine) for 14 days, affected extracellular L-glutamate level in the OFC [F_time_(9,90) = 53.7 (*p* < 0.01), F_administration_(1,10) = 7.4 (*p* < 0.01), F_adminisdtration*time_(9,90) = 12.5 (*p* < 0.01)] (Figure 7E,F). Perfusion with 100 μM AMPA into the MDTN increased extracellular L-glutamate level in the OFC (Figure 7E,F). Chronic guanfacine administration enhanced 100 μM AMPA-evoked dopamine release in the OFC (Figure 7E,F). Contrary to catecholamine, chronic administration of guanfacine did not affect basal L-glutamate level in the OFC (Figure 7E,F).

### 2.4. Effects of Subacute and Chronic Guanfacine Administration on α2A Adrenoceptor Expression in the Plasma Membrane of LC, OFC and VTA 

**Figure 8:**
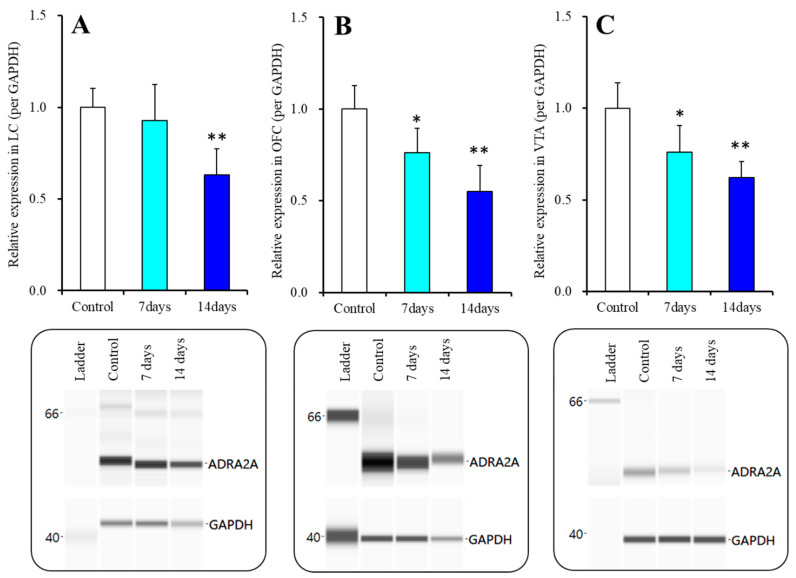
Effects of systemically subchronic (for 7 days: light blue columns) and chronic (for 14 days: blue columns) administrations of guanfacine on expression of α2A adrenoceptor in the plasma membrane of LC (Panel (**A**)), OFC (Panel (**B**)) and VTA (Panel (**C**)), using capillary immunoblotting system. Ordinate: mean ± SD (n = 6) of the relative protein level of α2A adrenoceptor (ADRA2A) in the plasma membrane fraction. Effects of guanfacine on α2A adrenoceptor expression were analysed by one-way ANOVA with Tukey’s post hoc test (* *p* < 0.05, ** *p* < 0.01 vs. Control). The pseudo-gel images, using capillary immunoblotting were represented in the lower panels.

After the systemically subchronic (for 7 days) and chronic (for 14 days) administration of guanfacine (0.12 mg/kg/days) using osmotic pump (N = 12), the total plasma membrane proteins were extracted using Minute Plasma Membrane Protein Isolation Kit (Invent Biotechnologies, Plymouth, MN, USA). The control was treated with vehicle (0.9% saline) for 14 days using osmotic pump. Subchronic (for 7 days) and chronic (for 14 days) guanfacine administrations time-dependently decreased α2A adrenoceptor expressions in the plasma membrane of LC [F(2,15) = 9.2, *p* < 0.01], OFC [F(2,15) = 20.6, *p* < 0.01] and VTA [F(2,15) = 13.6, *p* < 0.01], respectively (Figure 8). Expression of α2A adrenoceptor in the plasma membrane of LC was decreased by chronic (for 14 days) guanfacine administration, but not affected by subchronic (for 7 days) administration (Figure 8A). Contrary to LC, both subchronic (for 7 days) and chronic (for 14 days) decreased α2A adrenoceptor in the plasma membrane of OFC and VTA (Figure 8B,C).

## 3. Discussion

**Figure 9:**
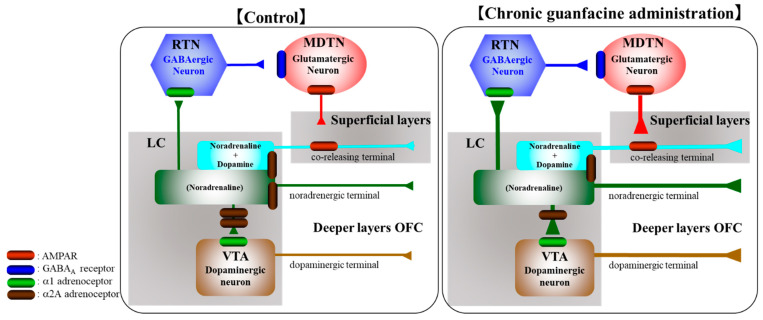
The proposed hypothesis for the catecholaminergic, noradrenergic, and co-releasing catecholaminergic (norepinephrine/dopamine) LC-OFC pathways, the intermediate glutamatergic LC-OFC (LC-RTN-MDTN-OFC) pathway, and the LC-VTA (LC-VTA-OFC) dopaminergic pathway. LC projects three afferents, including the selective norepinephrine releasing terminal, to deeper OFC (the direct noradrenergic pathway), RTN (a part of the intermediate glutamatergic pathway), VTA (a part of the intermediate dopaminergic pathway), and superficial OFC layers (the direct co-releasing catecholaminergic pathway). The co-releasing terminal in the OFC receives excitatory glutamatergic input from MDTN (a part of the intermediate glutamatergic pathway). The selective norepinephrine releasing terminal in RTN activates the GABAergic neuron in RTN, resulting in the inhibition of the MDTN glutamatergic neuron. The selective norepinephrine releasing terminal in the VTA activates the dopaminergic neuron in the VTA via the α1 adrenoceptor, resulting in increased dopamine release in the OFC. The acute activation of the postsynaptic/somatodendritic α2A adrenoceptor in the LC by guanfacine reduces noradrenergic transmission in noradrenergic LC-OFC and LC-VTA pathways and catecholaminergic transmission in the co-releasing catecholaminergic LC-OFC pathway. Contrary to the acute effect of guanfacine, chronic guanfacine administration enhances catecholaminergic transmission in both noradrenergic LC-OFC and LC-VTA pathways and catecholaminergic LC-OFC pathways via the downregulation of inhibitory α2A adrenoceptors.

Catecholaminergic neurons in the LC project catecholaminergic terminals to various brain regions, including the OFC, VTA and RTN [24,25,26,27,28,34,35,38]. Interestingly, the LC projects selective noradrenergic terminals to the deeper layers of the OFC (the mesocortical noradrenergic pathway) and co-releasing (dopamine and norepinephrine) terminals to the superficial layers of the OFC (the mesocortical co-releasing catecholaminergic pathway) [24,25,26,27,28,29,36]. For the other noradrenergic projections, the LC-VTA noradrenergic pathway activates dopaminergic transmission in the mesocortical (VTA-OFC) dopaminergic pathway via the excitatory postsynaptic α1 adrenoceptor [38]. Similar to the LC-VTA noradrenergic pathway, the LC-RTN noradrenergic pathway also activates intrathalamic (RTN-MDTN) GABAergic transmission via postsynaptic α1 adrenoceptor [24,29,34,35]. Therefore, the reduction in3 mesothalamic noradrenergic transmission enhances thalamocortical (MDTN-OFC) glutamatergic transmission due to intrathalamic GABAergic disinhibition. Interestingly, thalamocortical glutamatergic transmission selectively activates the mesocortical co-releasing catecholaminergic terminal in superficial layers of the frontal cortex but does not affect the mesocortical noradrenergic pathway [24,27,28,31,35,39]. Based on these previous findings, to explore the clinical action of guanfacine, the present study determined the chronic administration of therapeutically relevant doses of guanfacine on catecholaminergic transmission in the LC-OFC and LC-VTA, GABAergic inhibition in LC-RTN-MDTN, and thalamocortical glutamatergic transmission in MDTN-OFC. The catecholaminergic transmission pathway associated with the LC is represented in Figure 9. 

In our previous study, acute local administration into the LC and subchronic (0.12 mg/kg/day for 7 days) administrations of guanfacine decreased basal extracellular levels of dopamine and norepinephrine in the OFC [24,35], whereas the present study demonstrated that chronic administration of the therapeutically relevant dose [17,24,30] of guanfacine (0.12 mg/kg/day for 14 days) increased basal extracellular levels of dopamine and norepinephrine in the OFC and prevented the inhibitory effects of the local administration of guanfacine into the LC. The mechanisms of the time-dependent discrepant effects between acute, subchronic (for 7 days), and chronic (for 14 days) guanfacine administrations are probably induced by a combination of desensitization and downregulation of α2A adrenoceptor in the LC because the α2A adrenoceptor in the LC was downregulated by chronic (for 14 days) guanfacine administration but not by subchronic (for 7 days) administration (Figure 8). The α2A adrenoceptor in the LC is expressed in both presynaptic and postsynaptic/somatodendritic regions, which operates as an autoreceptor [24,35,40]. The α2A adrenoceptor is an inhibitory receptor that acts via coupling to the Gi protein, similar to the serotonin 5-HT1A receptor [41,42,43]. The chronic administration of selective serotonin reuptake inhibitors enhances serotonergic transmission via the downregulation of the serotonin 5-HT1A receptor [42,43]. Therefore, in the present results, the downregulation of the somatodendritic α2A adrenoceptor in the LC induced by chronic guanfacine administration generates the enhancement of catecholaminergic transmission via the attenuation of the function of the inhibitory α2A adrenoceptor. Contrary to chronic guanfacine administration, the subchronic administration of therapeutically relevant doses of guanfacine (for 7 days) generated weak desensitisation without the downregulation of the α2A adrenoceptor in the LC, resulting in the attenuation of basal extracellular catecholamine levels in the OFC. In other words, the function of the α2A adrenoceptor in the LC is attenuated but possibly remains functional during subchronic guanfacine administration [24].

Contrary to the LC, both subchronic and chronic guanfacine administration time-dependently downregulated the α2A adrenoceptor in the VTA. In the VTA, the α2A adrenoceptor is mainly expressed on the presynaptic region and suppresses norepinephrine release [38], whereas in our previous study, subchronic guanfacine administration weakly decreased basal extracellular dopamine levels in the OFC [24]. Released norepinephrine from noradrenergic terminals in the VTA enhances dopaminergic neurones in the VTA via the activation of the postsynaptic α1 adrenoceptor. The downregulation of the presynaptic α2A adrenoceptor in the VTA activates the postsynaptic α1 adrenoceptor, leading to the activation of dopaminergic neurones due to an enhanced release of norepinephrine induced by the disinhibition associated with the presynaptic α2A adrenoceptor. However, the affinity of the α1 adrenoceptor is more than 10 times lower in terms of sensitivity compared to the α2A adrenoceptor [13]. Therefore, enhanced basal dopamine release in the OFC possibly requires the higher elevation of norepinephrine release than the threshold level for the α1 adrenoceptor, induced by downregulations of both the somatodendritic α2A adrenoceptor in the LC and presynaptic α2A adrenoceptor. A clinical study using a reinforcement-learning task with fMRI revealed that stimulants improved task performance in patients with ADHD (enhancement of reward-learning rates and the ability to differentiate optimal from non-optimal novel choices) via reduced VTA responses to novelty compared to the control [20]. Taken together with the clinical findings, the present results suggest that enhanced tonic dopaminergic transmission due to the rapid downregulation of the presynaptic α2A adrenoceptor induced by guanfacine contributes to the mechanisms of clinical action of guanfacine as an improvement in novelty processing.

Thalamocortical glutamatergic transmission in the MDTN-OFC pathway plays important roles in cognitive/perception functions, including sensory integration and executive function [23,42,44]. MDTN glutamatergic neurones receive inputs from the amygdala, cortical, and other subcortical regions associated with learning, memory, emotion, and perceptual integration [29,45,46]. Indeed, the model of MDTN lesions continued to respond to stimuli after being satiated with food rewards [47]. Therefore, the thalamocortical (MDTN-OFC) pathway plays important roles in maintaining flexible stimulus–reward associations and emotional processing [48]. In other words, the MDTN regulates the integration of emotional and sensory information to the OFC. Anatomical/functional have also emphasised that functional abnormalities associated with the MDTN are involved in the cognitive dysfunction of ADHD [21]. Glutamatergic neurones in the MDTN output glutamatergic signalling to the OFC through select/integrate various sensory/emotional inputs to the MDTN [24,33,35,39,49,50,51,52,53,54]. The impairment of sensory/emotional integration in the MDTN is generated by two types of functional abnormalities—the elevation of the threshold for input signalling (less sensitivity to signalling inputs) and the desensitisation of phasic output signalling due to tonic hyperactivation. N-methyl-D-aspartate (NMDA)/glutamate receptor impairment schizophrenia models exhibit hyperactivation of thalamocortical glutamatergic transmission, which is compensated by several atypical antipsychotics, clozapine, aripiprazole and lurasidone [33,49,50,51,52,53,54]. In contrast, hypo-glutamatergic transmission in the thalamocortical pathway was observed in the genetic rodent model of autism [39,55,56,57,58]. A clinical study using fMRI reported that ketamine (an NMDA/glutamate receptor antagonist) suppressed functional LC-MDTN connectivity [59] through intrathalamic GABAergic disinhibition [23]. Although further studies addressing how the LC-RTN-MDTN pathway is involved in the pathophysiology and cognitive function of various neuropsychiatric disorders are needed, these clinical and preclinical findings emphasise that the LC-RTN-MDTN pathway plays important roles in the efficient switching between hippocampus-dependent and hippocampus-independent learning processes [60] leading to the regulation of attention and memory consolidation/reconsolidation processes [23,24,42,43,44,61,62].

Both acute guanfacine administration and chronic clonidine administration suppressed locomotion [17,63]. Therefore, both acute and chronic activations of the α2 adrenoceptor at least partially improve hyperactivity. In our previous study, the subchronic administration of therapeutically relevant doses of guanfacine (0.12 mg/kg/day for 7 days) enhanced thalamocortical (MDTN-OFC) glutamatergic transmission [24], and in the present study, chronic guanfacine administration (0.12 mg/kg/day for 14 days) also enhanced thalamocortical glutamatergic transmission; however, the mechanisms of enhanced thalamocortical glutamatergic transmission between acute, subchronic and chronic guanfacine administrations are not identical. Acute and subchronic guanfacine administrations improve phasic thalamocortical glutamatergic transmission, since both acute and subchronic guanfacine administrations lead to intrathalamic (RTN-MDTN) GABAergic disinhibition via reductions in mesothalamic (LC-RTN) noradrenergic transmission [24,35]. In contrast, chronic guanfacine administration also phasically enhanced thalamocortical glutamatergic transmission without affecting intrathalamic GABAergic transmission. Voltage-dependent sodium-channel-inhibiting antiepileptic drugs did not aggravate symptoms, whereas the AMPA/glutamate receptor inhibitor perampanel led to a high incidence of hostile and aggressive reactions [64]. Therefore, the acute and chronic administration of guanfacine improves the integration of sensory/emotional inputs in the MDTN, resulting in the enhancement of phasic glutamatergic output in the thalamocortical pathway. The present study could not explain the detailed mechanisms of these incomprehensible effects of chronic guanfacine administration on thalamocortical glutamatergic transmission. Both GABAergic neurones in the RTN and glutamatergic neurones in the MDTN receive a number of signals associated with GABA, L-glutamate, acetylcholine, noradrenaline, dopamine and serotonin [23,24,33,35,39,42,44,51,52,53,61,62]. The stimulatory effects of chronic guanfacine administration on thalamocortical glutamatergic transmission are probably mediated by combination with various transmission systems. To clarify this process, further studies are needed. The consistent enhancement of thalamocortical glutamatergic transmission by guanfacine, even if the mechanisms among acute, subchronic and chronic administrations are not identical, can reasonably explain the rapid onset of the clinical effects of guanfacine.

Both atomoxetine (a selective norepinephrine transporter inhibitor) and guanfacine (a selective α2A adrenoceptor agonist) are approved for the treatment of ADHD as non-stimulant class medications in Japan [7,24]. Recent meta-analyses and systematic reviews revealed the efficacies of both stimulant and non-stimulant class medications on the management of ADHD symptoms [7,65,66,67]; however, guanfacine displays a faster onset of improvement in ADHD symptoms compared to atomoxetine [7]. Indeed, one double-blind/placebo-controlled clinical trial demonstrated that guanfacine and atomoxetine displayed effectiveness onsets within 1 and 3 weeks, respectively [7]. Notably, another meta-analysis study reported that atomoxetine exhibited incidences associated with suicidal ideation within one month of starting treatment [9], whereas there are no reports of the risk of suicide-related events with guanfacine [7,10]. Therefore, the long-term effectiveness of atomoxetine and guanfacine are considered to be almost equal in the treatment of ADHD [1,2,3,4], whereas during the initiation of treatment with non-stimulant class medication, guanfacine is held in high esteem due to its rapid onset and fewer reports of serious adverse reactions compared to atomoxetine [7,9,10]. Furthermore, compared with atomoxetine, guanfacine probably provides greater control of certain ADHD symptoms, such as hypermotor and oppositional symptoms [7,8].

Noradrenergic transmission in the OFC is considered to be involved in fostering stability and flexibility against environmental changes [68]. The persistent reduced noradrenergic transmission in the OFC generates robust stability and remains in an initial state, but does not respond well to critical changes, whereas conversely, the persistent enhanced noradrenergic transmission found in the OFC generates quite unstable and cycling concentrations in response to a sequence of environmental changes [68]. It has also been established that mesocortical dopaminergic transmission is critically involved in modulating the neural circuits responsible for motivation associated with adaptive responses to rewarding and aversive events [69,70]. Therefore, mesocortical dopaminergic transmission also contributes to stability and flexibility against escape/avoidance events via a combination of suppressing inputs, which does not require immediate action with amplifying inputs associated with escape/avoidance events [71].

Atomoxetine acutely and chronically (for 21 days) enhanced frontal norepinephrine release, whereas atomoxetine-induced norepinephrine release was reduced by chronic atomoxetine administration [72]. Considering the clinical findings that three weeks is needed for the onset of the efficacy of atomoxetine, the downregulation of adrenoceptor probably contributes to the clinical action of atomoxetine. Therefore, the drastic enhanced noradrenergic transmission taking place until the downregulation of the adrenoceptor is induced by atomoxetine may provide unstable and cycling concentrations in response to a sequence of environmental changes. In contrast, during the initial stage, guanfacine selectively activates the α2A adrenoceptor, since guanfacine decreases norepinephrine release until the adrenoceptor is downregulated. Additionally, the enhancement of thalamocortical glutamatergic transmission during the initiation and chronic continuation of medication with guanfacine plays an important role in the rapid onset of the clinical efficacy of guanfacine. Taken together with previous clinical and preclinical findings, the present results regarding the effects of guanfacine on basal extracellular catecholamine levels in the OFC suggest that a combination of the direct activation of the α2A adrenoceptor with a reduction in basal releases of norepinephrine and dopamine in the OFC induced by guanfacine play important roles in the mechanisms of the rapid onset of improvement in hypermotor and oppositional symptoms. Additionally, the enhanced basal extracellular levels of norepinephrine and dopamine in the OFC induced by chronic guanfacine administration also contribute to improvement in the attention deficits of ADHD due to the activation of noradrenergic regulation associated with flexibility and dopaminergic regulation associated with amplifying important information.

The present study extensively explored the effects of acute and chronic administration of guanfacine on noradrenergic transmission associated with LC. Other noradrenergic nuclei, nucleus tractus solitarii, and the parabrachial nucleus also project noradrenergic terminals to various subcortical regions, such as the amygdala and thalamic nuclei, affecting cognition and emotions via interactions with monoaminergic transmissions [73,74,75,76]. Guanfacine is a selective α2A agonist, but the mechanism of clinical action via the modulation of noradrenergic transmission is probably more complicated than we expect. Therefore, further studies are required to elucidate the detailed pathophysiology of ADHD.

## 4. Materials and Methods

### 4.1. Chemical Agents and Drug Administration

Guanfacine (selective α2A adrenoceptor agonist) was obtained from Cosmo Bio (Tokyo, Japan). Prazosin (PRZ, selective α1 adrenoceptor antagonist) and α-amino-3-hydroxy-5-methyl-4-isoxazolepropionic acid (AMPA, selective AMPA/glutamate receptor agonist) were obtained from Fujifilm-Wako (Osaka, Japan). 

All compounds were prepared on the day of experiments. All drugs were perfused in modified Ringer’s solution (MRS), 145 mM Na^+^, 2.7 mM K^+^, 1.2 mM Ca^2+^, 1.0 mM Mg^2+^, and 154.4 mM Cl^−^ adjusted to pH 7.4 using 2 mM phosphate buffer and 1.1 mM Tris buffer. For microdialysis study, guanfacine and AMPA were dissolved directly in MRS. PRZ was initially dissolved in dimethyl sulfoxide at 25 mM [77,78]. The final dimethyl sulfoxide concentration was lower than 0.1% (vol/vol).

To study the effects of systemically subchronic and chronic administrations of therapeutically relevant doses of guanfacine, male Sprague Dawley rats (7 weeks old; SLC, Shizuoka, Japan) (total N = 84) were treated for 7 or 14 days with or without guanfacine (0.12 mg/kg/day), using osmotic pumps (2002; Alzet, Cupertino, CA, USA) implanted subcutaneously in the dorsal region, under 1.8% isoflurane anaesthesia. All rats were weighed prior to initiation of the study [43,50,51,55]. Guanfacine was dissolved in saline at 10 mg/mL/kg body weight. After surgery, rats were housed individually in cages, with food and water ad libitum in a controlled environment (22 ± 1 °C) under a 12 h dark/12 h light cycle.

### 4.2. Preparation of Microdialysis

Animal care, experimental procedures, and protocols for the animal experiments were approved by the Animal Research Ethics Committee of the Mie University School of Medicine (No. 29–21, 7 April 2018). To determine the effects of guanfacine on catecholaminergic transmission, all rats were chronically (for 14 days) treated with 0.12 mg/kg/day guanfacine, since the continuous systemic administration of 0.12 mg/kg/day guanfacine for 7 days desensitizes the α2 adrenoceptor. 

Three days after osmotic pump removal (after systemically chronic administration for 14 days), rats were anesthetized with 1.8% isoflurane and then placed in a stereotaxic frame. Concentric direct insertion-type dialysis probes were implanted in the OFC (A = +3.2 mm, L = +2.4 mm, V = −6.5 mm, relative to bregma) (0.22 mm diameter, 2 mm exposed membrane; Eicom, Kyoto, Japan), MDTN (A = −3.0 mm, L = +0.6 mm, V = −6.4 mm, relative to bregma) (0.22 mm diameter, 2 mm exposed membrane; Eicom) at a lateral angle of 30°, VTA (A = −5.6 mm, L = +2.1 mm, V = −9.0 mm, relative to bregma; 0.22-mm diameter; 1.5 mm of exposed membrane; Eicom) or LC (A = −9.7 mm, L = +1.3 mm, V = −7.6 mm, relative to bregma) (0.22 mm diameter, 1 mm exposed membrane; Eicom) according to the atlas of Paxinos and Watson [35,49,50,51,52,54,55,79].

Following surgery, rats were individually housed in cages during recovery and the experiment, with food and water provided ad libitum. Perfusion experiments commenced 18 h after recovery from isoflurane anaesthesia. Rats were placed individually into the system for freely moving animals (Eicom), equipped with a two-channel swivel (TCS2-23; ALS, Tokyo, Japan). The perfusion rate was set at 2 μL/min in all experiments, using MRS. Dialysate was collected every 20 min. Extracellular levels of norepinephrine, dopamine and L-glutamate were measured 8 h after the commencement of perfusion [80,81]. The microdialysis experiments were carried out on awake and freely moving rats. To determine the effects of guanfacine, PRZ and AMPA, the perfusion medium was then switched from MRS to MRS containing the target agent. Each dialysate was injected into an ultra-high-performance liquid chromatography (UHPLC) system.

After the microdialysis experiments, the brain was removed after cervical dislocation during isoflurane overdose anaesthesia. The locations of the dialysis probes were verified using brain tissue slices by stereoscopic microscope (SZ60, Olympus, Tokyo, Japan).

### 4.3. Determination of Levels of Dopamine, Norepinephrine and L-Glutamate

The levels of L-glutamate were determined by UHPLC (PU-4285; Jasco, Tokyo, Japan) with fluorescence detection (FP-4025; Jasco) after dual derivatization with isobutyryl-L-cysteine and o-phthalaldehyde. Derivative reagent solutions were prepared by dissolving isobutyryl-L-cysteine (2 mg) and o-phthalaldehyde (2 mg) in 100 μL of ethanol followed by the addition of 900 μL of sodium borate buffer (0.2 M, pH 9.0). Automated pre-column derivatization was carried out by drawing up a 5 μL aliquot sample, standard or blank solution and 5 μL of derivative reagent solution and holding them in reaction vials for 5 min before injection. The derivatized samples (5 μL) were injected using an autosampler (AS-4150; Jasco). The analytical column (YMC Triart C18, particle 1.8 μm, 50 × 2.1 mm; YMC, Kyoto, Japan) was maintained at 45 °C and the flow rate was set at 500 μL/min. A linear gradient elution program was performed over 10 min with mobile phases A (0.05 M citrate buffer, pH 7.0) and B (0.05 M citrate buffer containing 30% acetonitrile and 30% methanol, pH 2.0). The excitation/emission wavelengths of the fluorescence detector were set at 280/455 nm [82,83,84,85,86].

The levels of norepinephrine and dopamine were determined by UHPLC (xLC3185PU; Jasco) and electrochemical detection (ECD-300; Eicom), with a graphite carbon electrode set at +450 mV (vs. Ag/AgCl reference electrode). The analytical column (Triart C18, particle 1.8 μm, 30 × 2.1 mm; YMC) was maintained at 25 °C and the flow rate of the mobile phase was set at 500 μL/min. The mobile phase was composed of 0.1 M acetate buffer containing 1% methanol and 50 mg/L EDTA-2Na; the final pH was 6.0 [35,37,87].

### 4.4. Capillary Immunoblotting Analysis

Total plasma membrane proteins of LC, OFC and VTA were extracted using Minute Plasma Membrane Protein Isolation Kit (Invent Biotechnologies, Plymouth, MN, USA). The capillary immunoblotting analyses were performed using Wes (ProteinSimple, Santa Clara, CA, USA) according to the mainly ProteinSimple user manual. The lysates of plasma membrane fraction were mixed with a master mix (ProteinSimple) to a final concentration of 1 × sample buffer, 1 × fluorescent molecular weight marker, and 40 mM dithiothreitol and then heated at 95 °C for 5 min. The samples, blocking reagents, primary antibodies, HRP-conjugated secondary antibodies, chemiluminescent substrate (SuperSignal West Femto: Thermo Fisher Scientific, Waltham, MA, USA), and separation and stacking matrices, were also dispensed to the designated wells in a 25-well plate. After plate loading, the separation electrophoresis and immunodetection steps took place in the capillary system and were fully automated. A capillary immunoblotting analysis was carried out at room temperature, and the instrument’s default settings were used. Capillaries were first filled with a separation matrix followed by a stacking matrix, with about 40 nL of the sample used for loading. During electrophoresis, the proteins were separated by molecular weight through the stacking and separation matrices at 250 volts for 40–50 min and then immobilized on the capillary wall using proprietary photo-activated capture chemistry. The matrices were then washed out. The capillaries were next incubated with a blocking reagent for 15 min, and the target proteins were immunoprobed with primary antibodies followed by HRP-conjugated secondary antibodies (Anti-Rabbit IgG HRP, A00098, 10 μg/mL, GenScript, Piscataway, NJ, USA). The antibodies of ADRA2A (14266-1-AP, 1:100, Proteintech, Rosemont, IL, USA) and GAPDH (NB300-322, 1:100, Novus Biologicals, Littleton, CO, USA) were diluted in an antibody diluent (ProteinSimple) [50,55,82,83].

### 4.5. Data Analysis

Where possible, we randomized and blinded sample data. To determine extracellular transmitter levels and α2A adrenoceptor expression, the sample order was set on the autosampler according to a random number table. Drug doses and sample sizes were selected according to previous studies. All experiments in this study were designed with equally sized animal groups (N = 6) and all values were expressed as means ± standard deviation (SD). Differences were considered significant when *p* < 0.05 (two-tailed).

Regional transmitter concentrations were analysed using Mauchly’s sphericity test followed by multivariate analysis of variance (MANOVA) using BellCurve for Excel ver. 3.20 (Social Survey Research Information Co., Ltd., Tokyo, Japan). The data were composed of average of pre-treatment period (1 point) and each time point during target agent administration (9 points). When the data did not violate the assumption of sphericity (*p* > 0.05), the F-value of MANOVA was analysed using sphericity-assumed degrees of freedom. When the assumption of sphericity was violated (*p* < 0.05), F-values were analysed using Chi–Muller’s corrected degrees of freedom by BellCurve for Excel. When F-values for drug factors were significant in MANOVA, the data were finally analysed using Tukey’s post hoc test with BellCurve for Excel. Transmitter levels were expressed as the area under the curve between 20 and 180 min (AUC20–180 min) after target agent administration. All statistical analyses complied with the recommendations for experimental design and analysis in pharmacology. The protein expression of α2A adrenoceptor in the LC, OFC and VTA in the plasma membrane fraction was analysed by a one-way ANOVA with Tukey’s multiple comparison using BellCurve for Excel.

## 5. Conclusions

The present study demonstrated the time-dependent mechanisms of action of guanfacine. During acute guanfacine administration, guanfacine suppresses noradrenergic transmission from LC to OFC, VTA, and RTN due to the activation of the inhibitory postsynaptic/somatodendritic α2A adrenoceptor in the LC and presynaptic α2A receptor in the OFC, VTA and RTN. The co-releasing (norepinephrine with dopamine) pathway (from the LC to OFC) is also weakly suppressed by guanfacine, whereas thalamocortical glutamatergic transmission (MDTN-OFC) is enhanced via intrathalamic (RTN-MDTN) GABAergic disinhibition. In contrast with early-stage administration, chronic guanfacine administration (probably more than 2 weeks in duration) enhances noradrenergic transmission from the LC to OFC, VTA, and RTN due to the downregulation of the postsynaptic/somatodendritic and presynaptic α2A adrenoceptors. Furthermore, the attenuation of the inhibitory α2A adrenoceptor also produces an enhancement of the dopaminergic transmission in the LC-OFC co-releasing pathway and mesocortical (VTA-OFC) pathway. Unexpectedly, intrathalamic GABAergic transmission was not affected by acute guanfacine administration after chronic guanfacine administration, whereas thalamocortical glutamatergic transmission was enhanced via amplifying the integration of sensory/emotional inputs in the MDTN. These time-dependent actions of guanfacine, from the tonic-inhibition to tonic-activation of mesocortical catecholaminergic transmission, and phasic-activation associated with thalamocortical glutamatergic transmission support rapid onset and continuous efficacy in the treatment of ADHD.

## Data Availability

The data presented in this study are available on request from the corresponding author. The data are not publicly available due to equipment dependent data.
